# Supplementary material for: Evaluation of the Possibility of Using Hydroponic Cultivations for the Removal of Pharmaceuticals and Endocrine Disrupting Compounds in Municipal Sewage Treatment Plants
Source: Molecules. 2019 Dec 31;25(1):162. doi: 10.3390/molecules25010162 (PMC6982867; doi:10.3390/molecules25010162)
Supplement: Supplementary file 1 [file molecules-25-00162-s001.pdf]

molecules

Supplementary Materials

Article

Evaluation of the possibility of using hydroponic cultivations for the removal of pharmaceuticals and endocrine disrupting compounds in municipal sewage treatment plants

Daniel Wolecki[1], Magda Caban[1], Magdalena Pazda[1], Piotr Stepnowski[1], Jolanta Kumirska[1*]

[1] Faculty of Chemistry, University of Gdańsk, ul. Wita Stwosza 63, 80-308 Gdansk, Poland

Received: date; Accepted: date; Published: date

Tables and Figures content:

Table S1 Literature data concerning on the studies of usefulness of hydroponically cultivated plants for removing target compounds from sewage stream

Table S2 Chemical structures and physical and chemical properties of selected non-steroidal anti-inflammatory drugs (NSAIDs), analgesics, β-blockers, β-agonists, antidepressant drugs and estrogen-based hormones

Figure S1 The mass spectra of target compounds with the MS fragments assignation

Figure S2 Activated sludge chamber with a system of constructed wetlands in the investigated Municipal Wastewater Treatment Plant in Sochaczew (Mazowieckie Voivodeship, Poland)

Figure S3 Example chromatogram with marked SIM ions for determined target compounds in real Papyrus (*Cyperus papyru*) samples

Figure S4 Example chromatogram with marked SIM ions for determined target compounds in real Yellow pimpernel (*Lysimachia nemorum*) samples

Figure S5 Example chromatogram with marked SIM ions for determined target compounds in real European spindle (*Euonymus europaeus*) samples

Table S1 Literature data concerning on the studies of usefulness of hydroponically cultivated plants for removing target compounds from sewage stream

Pharmaceuticals/EDCs	Type of CW / laboratory system / full-scale system in WWTP	Plant	Method of determining target compounds in plant material			MQL [ng/g d.w.]	Concentration in plant material [ng/g d.w.]	Concentration in untreated sewage [ng/L]	Concentration in treated sewage [ng/L]	Elimination efficiency from wastewater stream (EE%)	Ref.
			Extraction	Clean-up	Detection						
18 analytes e.g. Ibuprofen Ketoprofen Naproxen Diclofenac Salicylic acid Caffeine Carbamazepine	*Mesocosm-scale*	*Typha angustifolia Phragmites australis*	Only roots ASE acetone/hexane, 1:1; v/v; two extraction cycles of 13.5 min at a temperature of 104 °C	Clean-up A florisil column with different elution of the neutral/acidic fractions. Neutral fraction directly analysed, and the acidic fraction derivatized with TMSH	GC-MS/MS	17 20 29 30 19 31 22	Detail in Fig. 3 Ibuprofen Salicylic acid Caffeine detected Ketoprofen Naproxen Diclofenac Carbamazepine not detected Salicylic acid - the main substance detected in root tissues (123-2560 ng/g)	Detail in Table 1 in the range 250-19 000	Detail in Table 1 in the range 10 -5740	*T. angustifolia* a better performance	[27]
Paracetamol	*Pilot scale*	*Phragmites australis Typha latifolia*	Not detected in plant material			-	nd	Fig. 3.	Fig. 3.	The P*hragmites* bed 51.7% for a Hydraulic Loading Rate (HLR) of 240	[29]

										mm/d to 87% with 120 mm/d HLR and 99.9% with 30 mm/d. The *Typha* bed a similar behavior with percentages of removal slightly lower, ranging from 46.7% (HLR of 240 mm/d) to >99.9% (hydraulic loading rate of 30 mm/d). At the same HLR values the unplanted bed removed between 51.3% and 97.6%	
86 pharmaceuticals e.g. Diclofenac Ibuprofen Ketoprofen Naproxen Alpha Ethinylestradiol	*Full-scan*	*Salix alba* *Iris pseudacorus* *Juncus effuses* *Callitriche palustris* *Carex caryophyllea*	20 g dry weight of each sample 1 L acetonitrile with 0.5% formic acid (v:v) under gentle	SPE SiO_2 cartridge system	UPLC-MS/MS		Ibuprofen was detected in all plant samples	in the range 1 ng/ L and 1000000 ng/L 6483 5004 604 22464 EE2 1140626	7377 3129 319 19904 EE2 901618	In detail Fig. 6. drug removal efficiency within the SFTW followed a seasonal trend, with the best	[32]

Beta Estradiol Estriol			mixing over a period of 24 h at 4 °C					- E3 17245	- E3 2860	results occurring in the summer	
8 Compounds, e.g. Caffeine Carbamazepine Ibuprofen Fluoxetine Gemfibrozil	*Full-scan*	*Typha angustifolia*	The root and shoot samples anhydrous sodium sulfate and methanol; UAE 35°C for 15 min. and then centrifuged at 3500 rpm for 5 min.	SPE	LC-MS/MS	nd	Bioconcentration factors (BCFs) in *Typha angustifolia,* ranged between 60 to 2000 Up to several hundred ng/g for caffeine	Detail in Figs. 1 and 2	Detail in Figs. 1 and 2	Between -1588% and 95.1%	[33]
Sulfamethoxazole Atenolol Dilantin Carbamazepine Diazepam Diclofenac Naproxen Triclosan	Full-scale	*Typha sp.*	MAE	SPE (nd)	LC-MS/MS	nd	<2.5 <2.5 <10 <5.0 <5.0 <2.5 <5.0 <10	nd	in the range 0-900	Atenolol/Carbamazepine/Diclofenac/Triclosan 60–100% Dilantin/Diazepam/Sulfamethoxazole 0-60%	[34]
Triclosan	Pilot-scale	*Ceratophyllum demersum Lemna sp. Paspalum spp. Pontederia cordata Potamageton spp.*	QuEChERS 30 mL 1:1 hexane:ethyl acetate	The lipid clean-up	GC-MS	MDL: 17 (shoot) 6 (root)	26 Triclosan readily accumulates in the root tissues of free living	-	-		[35]

		Sagittaria graminea<br>*Typha sp.*					wetland plants and show species specific differences				
Diclofenac	Laboratory system	*Typha latifolia*	QuEChERS 1 ml 0.1 M HCl:ACN (1:1, v/v)	SPE, StrataX 30 mg	LC-MS	nd	0.2% of the initial amount of diclofenac (1 mg/L) detected in roots and leaves during one week exposure	nd	nd	nd	[36]
65 pharmaceuticals e.g.<br>Diclofenac<br>Ibuprofen<br>Naproxen	Full-scale	*Ceratophyllum demersum*<br>*Elodea spp.*<br>*Glyceria maxima*<br>*Myriophyllum spicatum*<br>*Nymphaeaceae*<br>*Schoenoplectus lacustris*<br>*Typha sp.*	Not detected in plant material			-	-	380 – 510<br>660 – 1500<br>64 – 290	290 – 350<br>80 – 740<br>16 - 190	24 – 36<br>5 – 88<br>34 – 75<br>(estimated removal rates)	[40]
Carbamazepine<br>Clofibric acid<br>Ibuprofen	Microcosm-scale	*Typha sp.*	Not detected in plant material			-	-	nd	nd	97<br>75<br>96	[41]
Carbamazepine<br>Clofibric acid<br>Diclofenac<br>Ibuprofen<br>Ketoprofen	Full-scale	*Phragmites australis*<br>*Typha latifolia*	Not detected in plant material			-	-	370<br>70<br>1250<br>40<br>2100<br>340	23<br>46<br>188<br>2<br>42<br>95	39<br>34<br>85<br>96<br>98<br>72	[42]

Naproxen									
Carbamazepine<br>Clofibric acid<br>Diclofenac<br>Flunixin<br>Ibuprofen<br>Ketoprofen<br>Naproxen	Full-scale	*Phragmites australis*<br>*Typha latifolia*	Not detected in plant material	-	-	370<br>70<br>1250<br>1060<br>40<br>2100<br>340	-	30-47<br>32-36<br>73-96<br>0-64<br>95-96<br>97-99<br>52-92	[43]
Caffeine<br>Diclofenac<br>Ibuprofen<br>Naproxen<br>Triclosan<br>Clofibric acid	Microcosm-scale	*Salvinia molesta*<br>*Lemna minor*<br>*Ceratophyllum demersum Elodea canadensis*	Not detected in plant material	-	-	nd	nd	81-99<br>99<br>44-77<br>40-53<br>96-99<br>16-23	[44]
73 target pharmaceuticals and e.g.<br>Paracetamol<br>Diclofenac<br>Ibuprofen<br>Naproxen<br>Nadolol	Pilot-scale	*Phragmites australis*	Not detected in plant material	-	-	30<br>284<br>81<br>178<br>< LOD	16<br>271<br>58<br>114<br>< LOD	98<br>38<br>94<br>86<br>100	[45]
Paracetamol<br>Diclofenac<br>Ibuprofen	*Mesocosm-scale*	*Phragmites australis*	Not detected in plant material	-	-	1500 – 34000<br>1900 – 2800<br>39900 – 83900	6390<br>760<br>18110	>95<br>32 - 70<br>52 - 85	[46]
Cotinine<br>Caffeine<br>Fluoxetine<br>Paracetamol<br>Naproxen<br>Ibuprofen	*Full-scale*	*Phragmites australis*<br>*Hydrocottle spp.*	Not detected in plant material	-	-	1097<br>25567<br>-<br>39300<br>10418<br>9922	12<br>28<br>-<br>10<br>90<br>38	>99<br>>99<br>-<br>>99<br>>99<br>>99	[47]

Gemfibrozil<br>Atenolol<br>Nadolol<br>Propranolol<br>Metoprolol<br>Sotalol						1652<br>1442<br>30<br>-<br>211<br>174	600<br>99<br>7<br>-<br>17<br>121	95<br>>99<br>>99<br>-<br>>99<br>82	
Ketoprofen<br>Naproxen<br>Ibuprofen<br>Diclofenac<br>Salicylic acid<br>Carbamazepine<br>Caffeine	*Mesocosm-scale*	*Typha angustifolia*<br>*Phragmites australis*	Not detected in plant material	-	-	1790; <890<br>3530; 1350<br>24190; 8380<br>830; 370<br>9930; 10290<br>1360; 1520<br>67340; 22590	nd	Winter / Summer<br>33-50 / 100<br>27-66 / 27-83<br>51-54 / 85-96<br>17 – 52<br>76-85 / 87-89<br>24-36/ 48<br>58-65 / 99<br>*P. australis* a better performance	[48]
Ketoprofen<br>Naproxen<br>Ibuprofen<br>Diclofenac<br>Salicylic acid<br>Carbamazepine<br>Caffeine	*Mesocosm-scale*	*Typha latifolia*<br>*Salix atrocinerea*	Not detected in plant material	-	-	Table 1 concentrations at each sampling point	<890<br>160<br>990<br>430<br>1650<br><1250<br>3570	77 – 81<br>73 – 85<br>42 – 99<br>65 – 87<br>94 – 97<br>-<br>83 - 96	[49]
Atenolol<br>Caffeine<br>Carbamazepine<br>Diclofenac<br>Glimepriride	Full-scale	*Acorus spp.*<br>*Typha sp.*	Not detected in plant material	-	-	nd	nd	Presented in Fig. 2<br>Compounds with greater hydrophilicity could be more	[50]

Ibuprofen Naproxen Sulfamethoxazole								efficiently removed than hydrophobic compounds	
Carbamazepine Diclofenac Ibuprofen Naproxen	Pilot-scale A Membrane Biological Reactors (MBR) and an Activated Sludge (AS) unit, combined with vertical flow reed beds (VFRB)	nd	Not detected in plant material	-	-	VFRB AS / VFRB MBR<br>nd / nd<br>nd / nd<br>3110 / 800<br>1810 / 840	nd / nd<br>nd / nd<br>530 / 90<br>270 / 150	Both systems reduced the discharge of micropollutants at comparable rates, ranging from 0.3 to 0.9 ppb for acidic drugs. A green emerging technology used for post-treatment, filtration through vertical flow reed-beds, led to a general improvement of effluent quality	[51]
Diclofenac Ibuprofen Naproxen	*Pilot-scale*	*Phragmites australis*	Not detected in plant material	-	-	20 – 260<br>23600 - 46800<br>1530 – 3940	nd	97-99	[52]

nd – Not described in detail in cited literature

References (position identical as in the manuscript)

27 Hijosa-Valsero, M.; Reyes-Contreras, C.; Domínguez, C.; Bécares, E.; Bayona, J.M. Behaviour of pharmaceuticals and personal care products in constructed wetland compartments: Influent, effluent, pore water, substrate and plant roots. *Chemosphere* **2016**, *145*, 508–517.

29 Ranieri, E.; Verlicchi, P.; Young, T.M. Paracetamol removal in subsurface flow constructed wetlands. *J. Hydrol.* **2011**, *404*, 130–135.

32 Nuel, M.; Laurent, J.; Bois, P.; Heintz, D.; Wanko, A. Seasonal and ageing effect on the behaviour of 86 drugs in a full-scale surface treatment wetland: Removal efficiencies and distribution in plants and sediments. Sci. Total Environ. 2018, 615, 1099–1109.
33 Wang, Y.; Yin, T.; Kelly, B.C.; Gin, K.Y.-H. Bioaccumulation behaviour of pharmaceuticals and personal care products in a constructed wetland. *Chemosphere* **2019**, *222*, 275–285.
34 Park, N.; Vanderford, B.J.; Snyder, S.A.; Sarp, S.; Kim, S.D.; Cho, J. Effective controls of micropollutants included in wastewater effluent using constructed wetlands under anoxic condition. Ecol. Eng. 2009, 35, 418–423.
35 Zarate, F.M.; Schulwitz, S.E.; Stevens, K.J.; Venables, B.J. Bioconcentration of triclosan, methyl-triclosan, and triclocarban in the plants and sediments of a constructed wetland. *Chemosphere* **2012**, *88*, 323–329.
36 Bartha, B.; Huber, C.; Schröder, P. Uptake and metabolism of diclofenac in Typha latifolia – How plants cope with human pharmaceutical pollution. *Plant Sci.* **2014**, *227*, 12–20.
40 Breitholtz, M.; Näslund, M.; Stråe, D.; Borg, H.; Grabic, R.; Fick, J. An evaluation of free water surface wetlands as tertiary sewage water treatment of micro-pollutants. *Ecotoxicol. Environ. Saf.* **2012**, *78*, 63–71.
41 Dordio, A.; Carvalho, A.J.P.; Teixeira, D.M.; Dias, C.B.; Pinto, A.P. Removal of pharmaceuticals in microcosm constructed wetlands using Typha spp. and LECA. *Bioresour. Technol.* **2010**, *101*, 886–892.
42 Llorens, E.; Matamoros, V.; Domingo, V.; Bayona, J.M.; García, J. Water quality improvement in a full-scale tertiary constructed wetland: Effects on conventional and specific organic contaminants. *Sci. Total Environ.* **2009**, *407*, 2517–2524.
43 Matamoros, V.; García, J.; Bayona, J.M. Organic micropollutant removal in a full-scale surface flow constructed wetland fed with secondary effluent. *Water Res.* **2008**, *42*, 653–660.
44 Matamoros, V.; Salvadó, V. Evaluation of the seasonal performance of a water reclamation pond-constructed wetland system for removing emerging contaminants. *Chemosphere* **2012**, *86*, 111–117.
45 Verlicchi, P.; Galletti, A.; Petrovic, M.; Barceló, D.; Al Aukidy, M.; Zambello, E. Removal of selected pharmaceuticals from domestic wastewater in an activated sludge system followed by a horizontal subsurface flow bed — Analysis of their respective contributions. *Sci. Total Environ.* **2013**, *454–455*, 411–425.
46 Ávila, C.; Reyes, C.; Bayona, J.M.; García, J. Emerging organic contaminant removal depending on primary treatment and operational strategy in horizontal subsurface flow constructed wetlands: Influence of redox. *Water Res.* **2013**, *47*, 315–325.
47 Conkle, J.L.; White, J.R.; Metcalfe, C.D. Reduction of pharmaceutically active compounds by a lagoon wetland wastewater treatment system in Southeast Louisiana. *Chemosphere* **2008**, *73*, 1741–1748.
48 Hijosa-Valsero, M.; Matamoros, V.; Sidrach-Cardona, R.; Martín-Villacorta, J.; Bécares, E.; Bayona, J.M. Comprehensive assessment of the design configuration of constructed wetlands for the removal of pharmaceuticals and personal care products from urban wastewaters. *Water Res.* **2010**, *44*, 3669–3678.
49 Hijosa-Valsero, M.; Matamoros, V.; Martín-Villacorta, J.; Bécares, E.; Bayona, J.M. Assessment of full-scale natural systems for the removal of PPCPs from wastewater in small communities. *Water Res.* **2010**, *44*, 1429–1439.
51 Reif, R.; Besancon, A.; Le Corre, K.; Jefferson, B.; Lema, J.M.; Omil, F. Comparison of PPCPs removal on a parallel-operated MBR and AS system and evaluation of effluent post-treatment on vertical flow reed beds. *Water Sci. Technol.* **2011**, *63*, 2411–2417.
50 Lee, S.; Kang, S.-I.; Lim, J.-L.; Huh, Y.J.; Kim, K.-S.; Cho, J. Evaluating controllability of pharmaceuticals and metabolites in biologically engineered processes, using corresponding octanol–water distribution coefficient. *Ecol. Eng.* **2011**, *37*, 1595–1600.
52 Ávila, C.; Pedescoll, A.; Matamoros, V.; Bayona, J.M.; García, J. Capacity of a horizontal subsurface flow constructed wetland system for the removal of emerging pollutants: An injection experiment. *Chemosphere* **2010**, *81*, 1137–1142.

Table S2 Chemical structures and physical and chemical properties of selected non-steroidal anti-inflammatory drugs (NSAIDs), analgesics, β-blockers, β-agonists, antidepressant drugs and estrogen-based hormones

Number	Trade name/CAS number	Function	pK_a	LogP	Chemical structure
**1**	Ibuprofen<br>15687-27-1	Analgesic/<br>NSAIDs	4.91	3.97	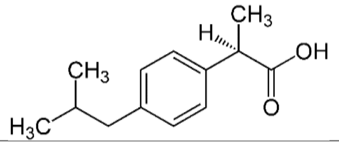
**2**	Paracetamol<br>103-90-2	Analgesic	9.38	0.46	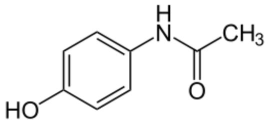
**3**	Flurbiprofen<br>5104-49-4	Analgesic/<br>NSAIDs	4.42[a]	4.16	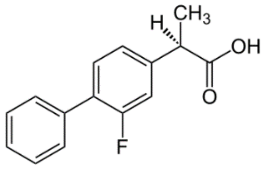
**4**	Naproxen<br>22204-53-1	Analgesic/<br>NSAIDs	4.15	3.18	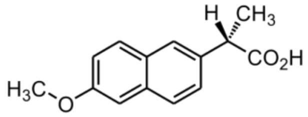
**5**	Diflunisal<br>22494-42-4	Analgesic/<br>NSAIDs	2.69[a]	4.44	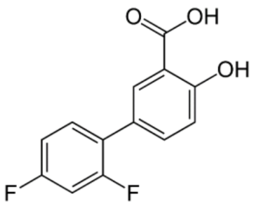
**6**	Diclofenac<br>15307-86-5	Analgesic/<br>NSAIDs	4.15	4.51	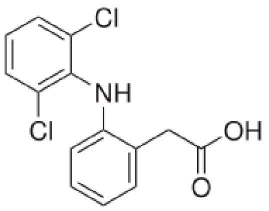

**7**	Nadolol<br>42200-33-9	β-blockers	9.67	0.81	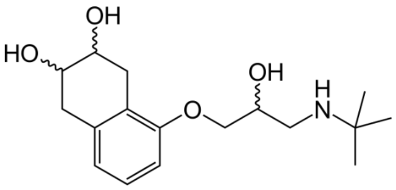
**8**	Terbutaline<br>23031-25-6	β_2-agonists	8.86[a]	0.90	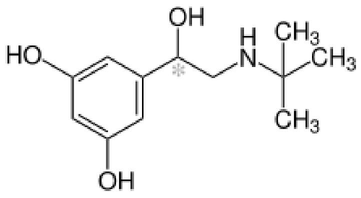
**9**	Amitriptyline<br>50-48-6	Antidepressant drugs/analgesic	9.40	4.92	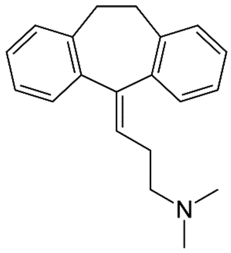
**10**	Imipramine<br>50-49-7	Antidepressant drugs	9.40	4.80	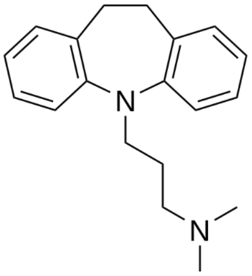
**11**	Clomipramine<br>303-49-1	Antidepressant drugs	9.20[a]	5.19	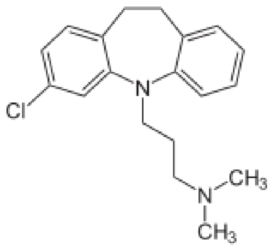

**12**	Estrone (E1)<br>53-16-7	Hormones	10.33[a]	3.13	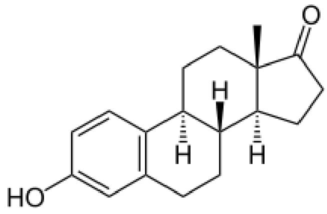
**13**	17β-estradiol (E2)<br>50-28-2	Hormones	10.33[a]	4.01	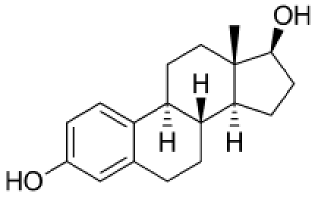
**14**	17α-ethinylestradiol (EE2)<br>57-63-6	Hormones	10.33[a]	3.67	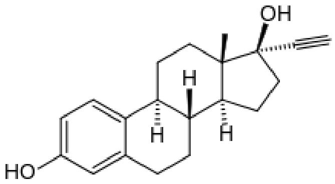
**15**	Estriol (E3)<br>50-27-1	Hormones	10.54	2.45	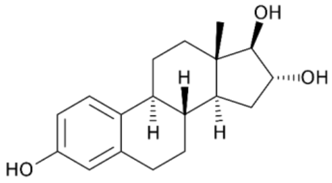

[a] Predicted property based on ChemAxon

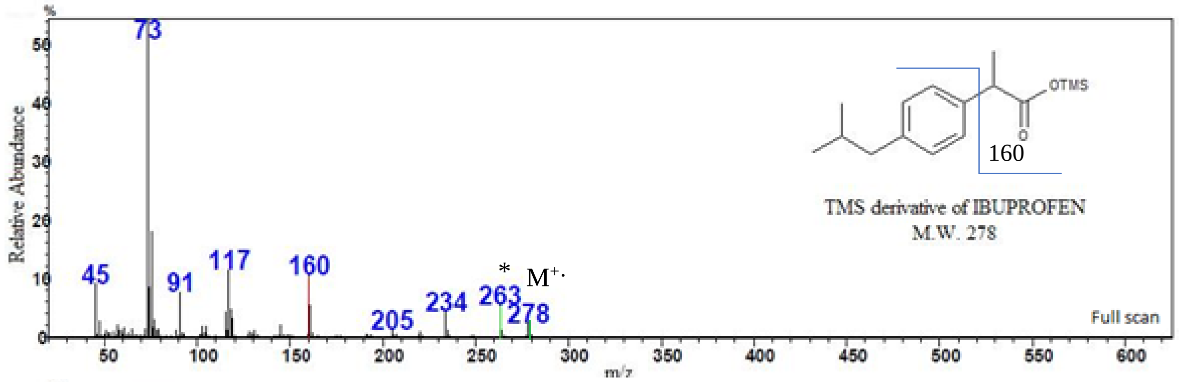
Relative Abundance
%
73
45
91
117
160
205
234
263
278
*
M+·
m/z
Full scan
OTMS
O
160
TMS derivative of IBUPROFEN
M.W. 278

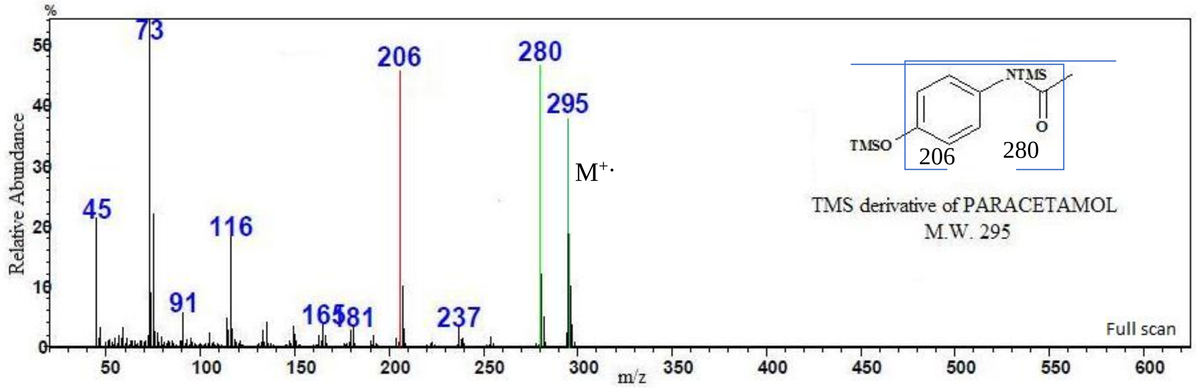
Relative Abundance
%
73
45
91
116
165
181
206
237
280
295
M+·
m/z
Full scan
NTMS
O
TMSO
206
280
TMS derivative of PARACETAMOL
M.W. 295

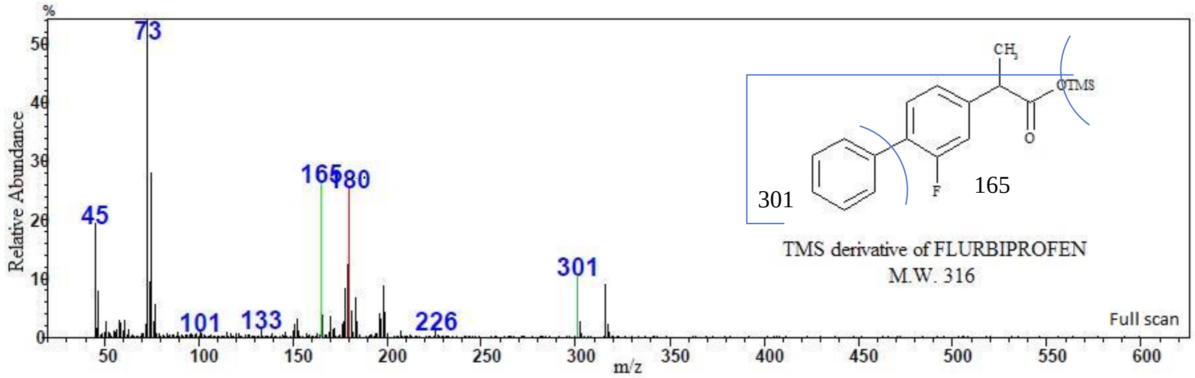
Relative Abundance
%
73
45
101
133
165
180
226
301
m/z
Full scan
CH3
OTMS
O
F
301
165
TMS derivative of FLURBIPROFEN
M.W. 316

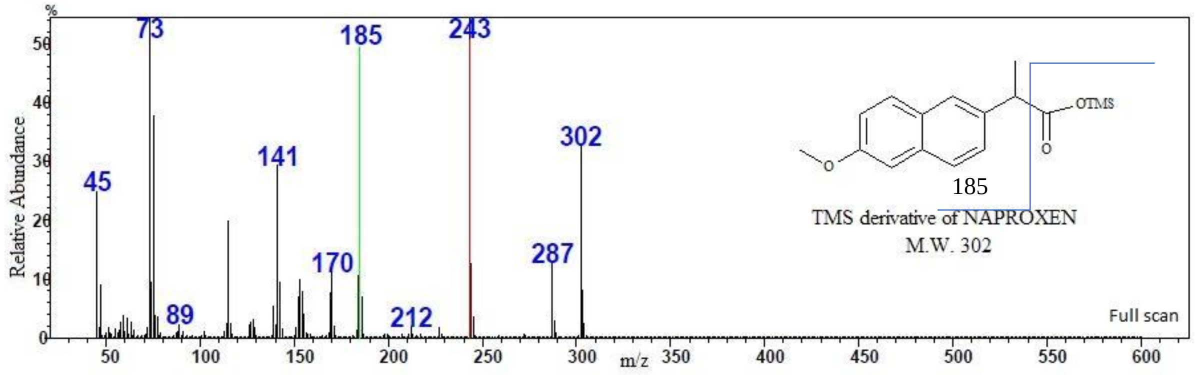
Relative Abundance
%
73
45
89
141
170
185
212
243
287
302
m/z
Full scan
OTMS
O
O
185
TMS derivative of NAPROXEN
M.W. 302

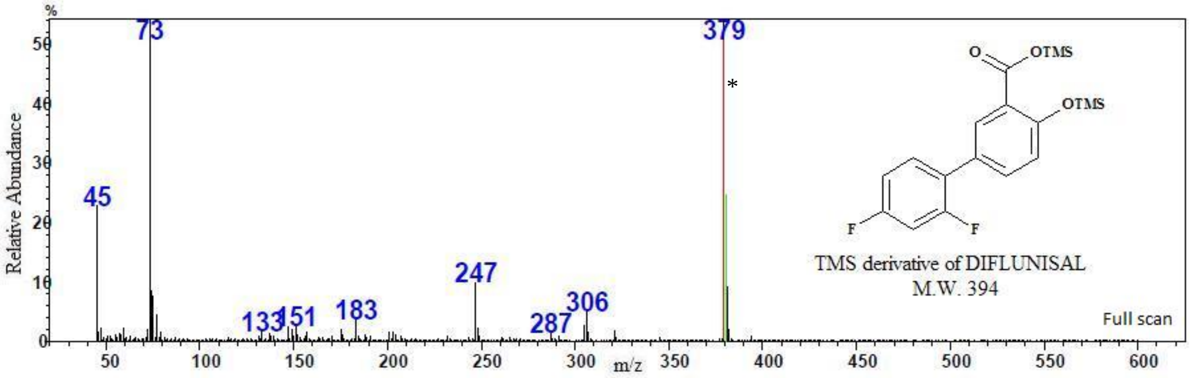

%
Relative Abundance
73
379
*
45
247
133
151
183
287
306
m/z
O
OTMS
OTMS
F
F
TMS derivative of DIFLUNISAL
M.W. 394
Full scan

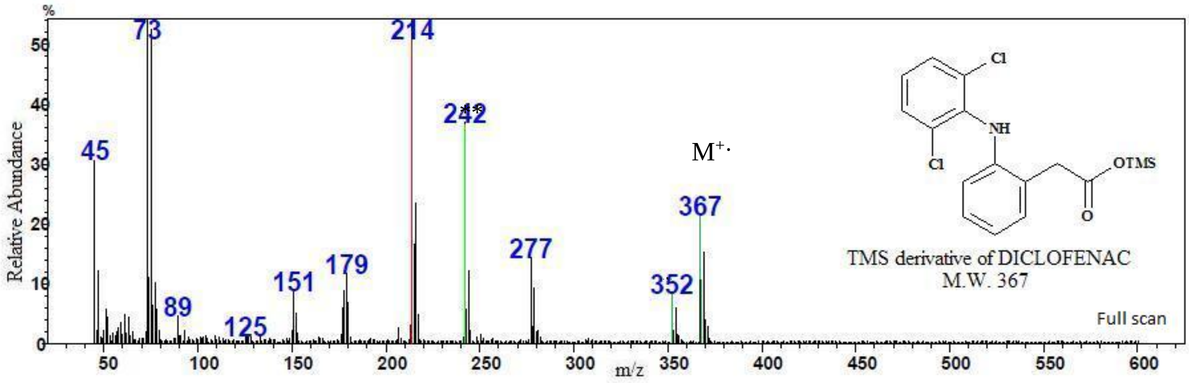

%
Relative Abundance
73
214
242
**
45
M+·
367
89
125
151
179
277
352
m/z
Cl
NH
Cl
OTMS
O
TMS derivative of DICLOFENAC
M.W. 367
Full scan

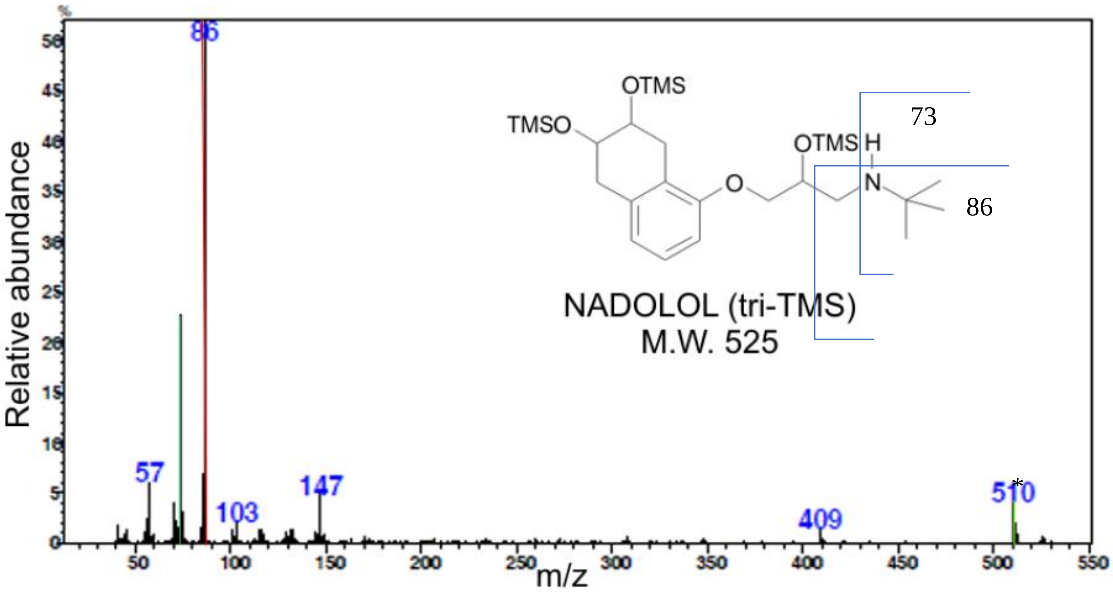

%
Relative abundance
86
57
103
147
409
510
*
m/z
OTMS
TMSO
OTMS
H
N
O
73
86
NADOLOL (tri-TMS)
M.W. 525

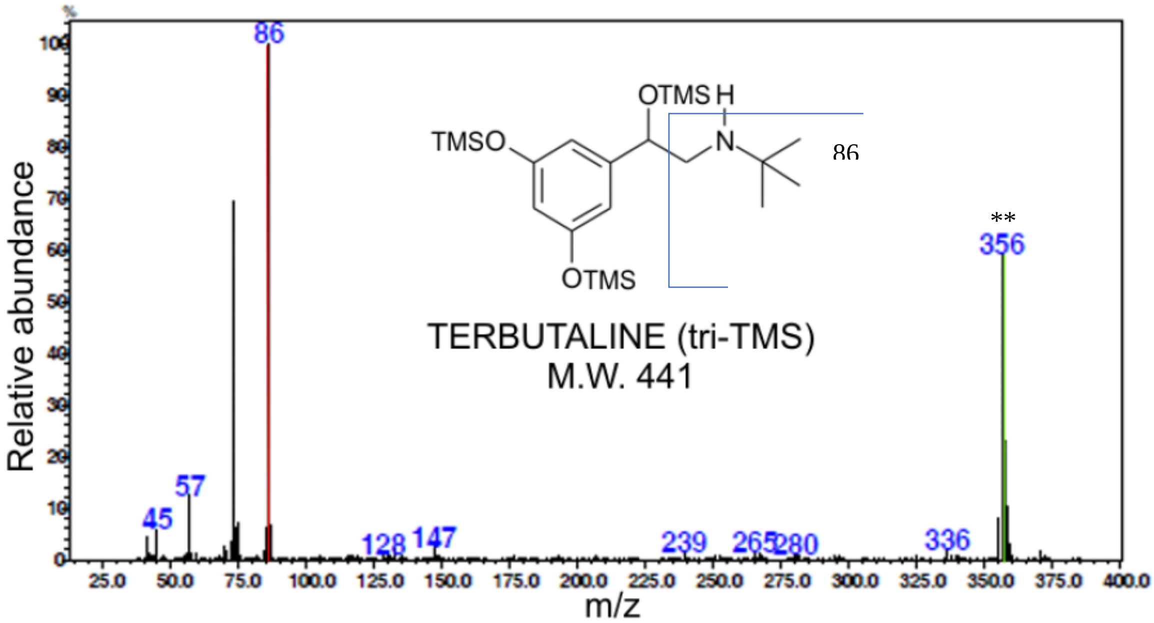
Relative abundance
m/z
86
57
45
128
147
239
265
280
336
**
356
OTMS H
TMSO
N
OTMS
86
TERBUTALINE (tri-TMS)
M.W. 441

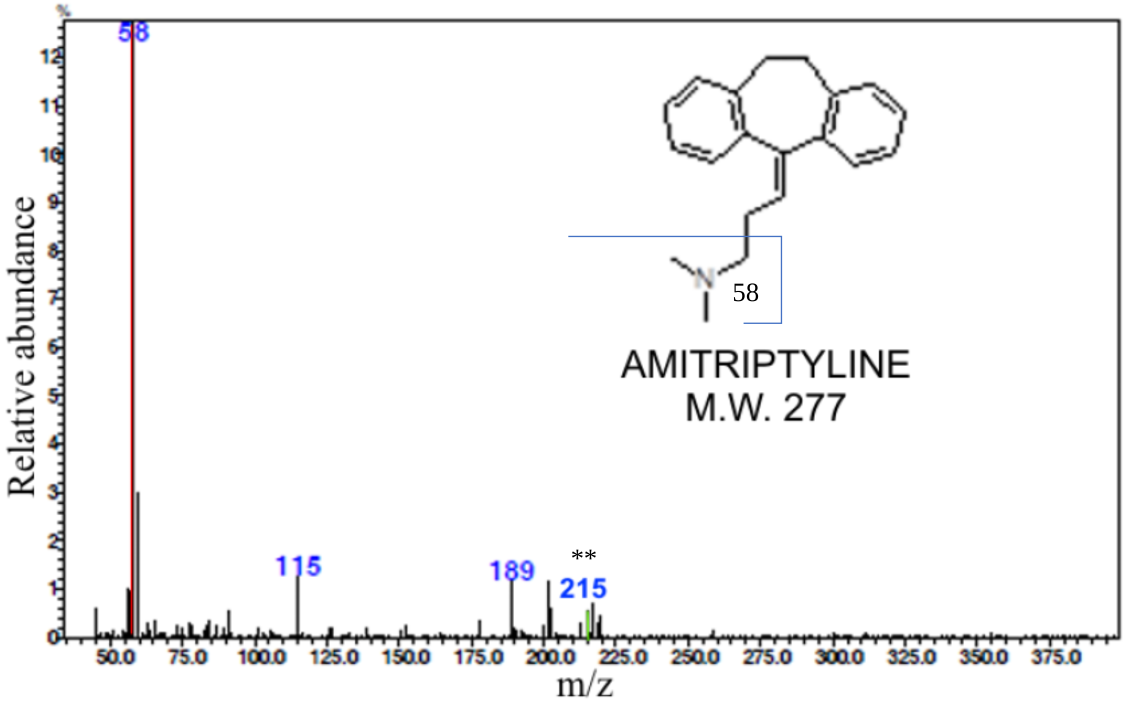
Relative abundance
m/z
58
115
189
**
215
N
58
AMITRIPTYLINE
M.W. 277

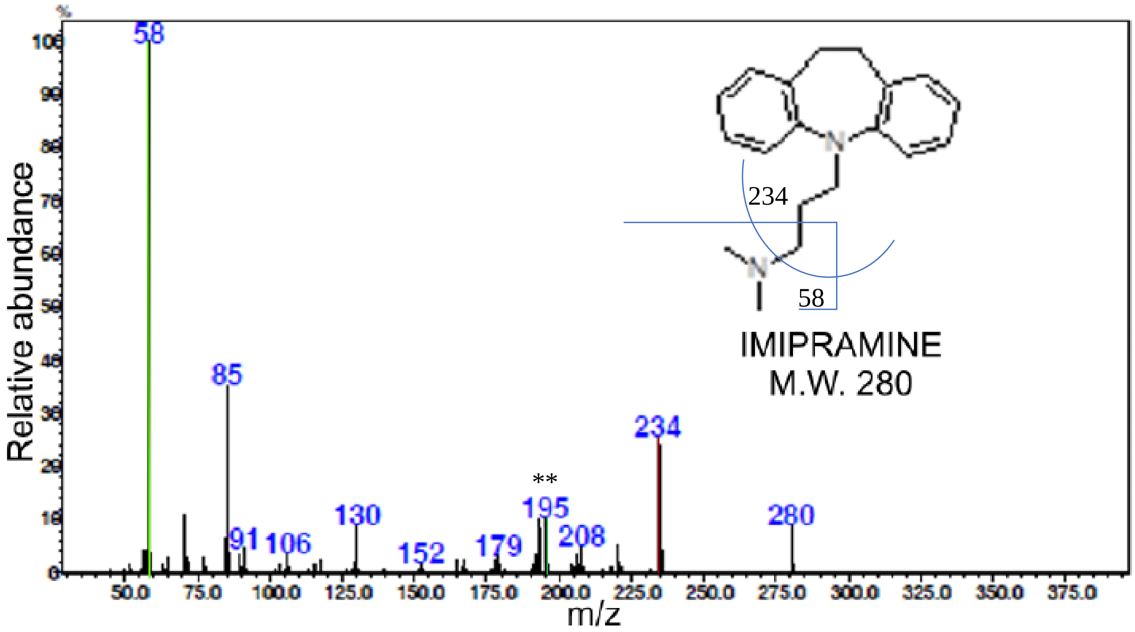
Relative abundance
m/z
IMIPRAMINE
M.W. 280
234
58
**
58
85
91
106
130
152
179
195
208
234
280

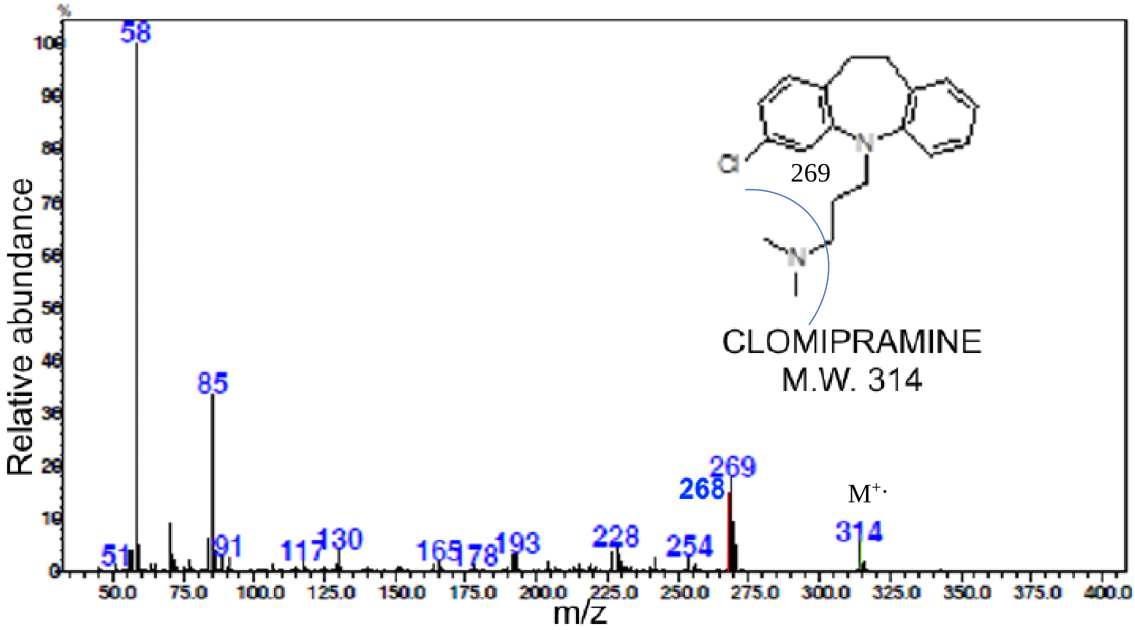
Relative abundance
m/z
CLOMIPRAMINE
M.W. 314
269
Cl
M+·
58
85
51
91
117
130
165
178
193
228
254
268
269
314

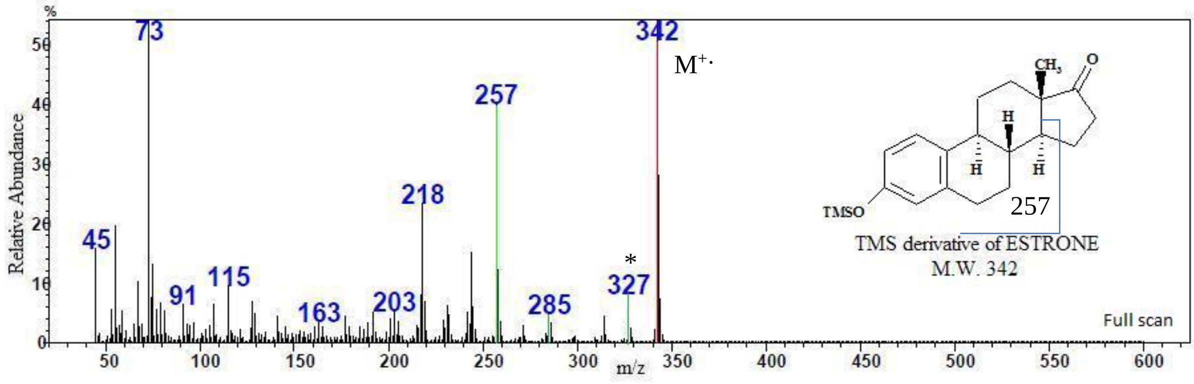
Relative Abundance
m/z
TMS derivative of ESTRONE
M.W. 342
TMSO
257
M+·
Full scan
73
342
257
218
45
91
115
163
203
285
*
327

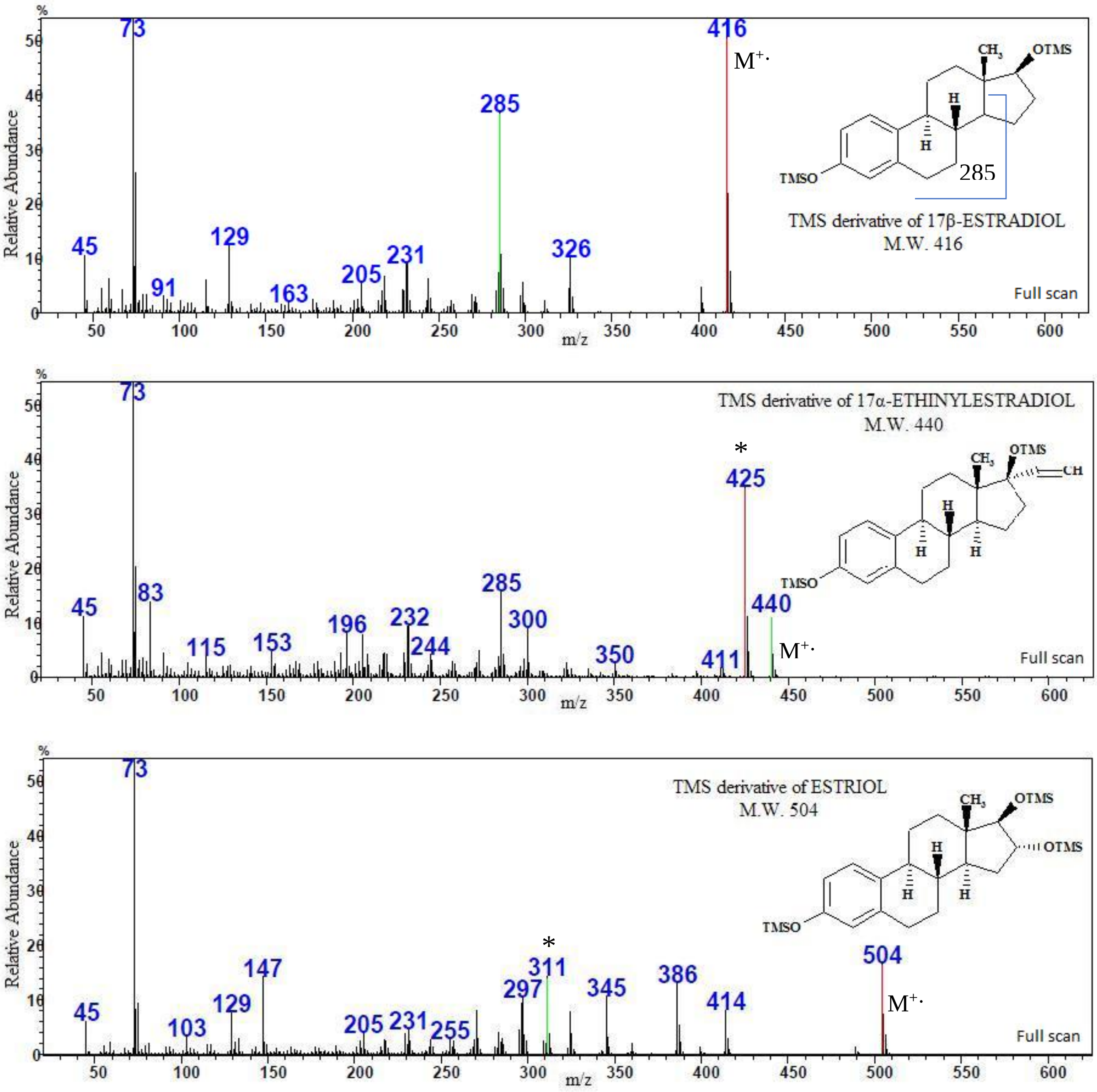

* ion [M-15] where 15 is derived from detachment of one CH_3 group from TMS

** The ion is formed in a complicated process of intramolecular rearrangement

Figure S1 The mass spectra of target compounds with the MS fragments assignation

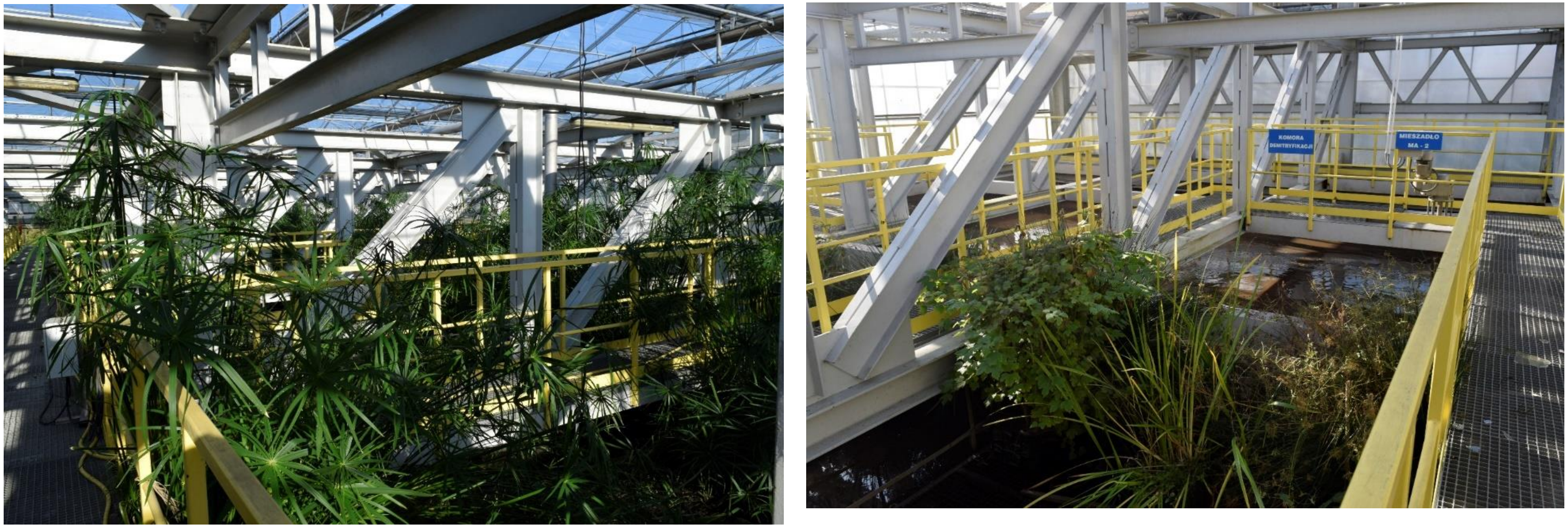

Figure S2 Activated sludge chamber with a system of constructed wetlands in the investigated Municipal Wastewater Treatment Plant in Sochaczew (Mazowieckie Voivodeship, Poland)

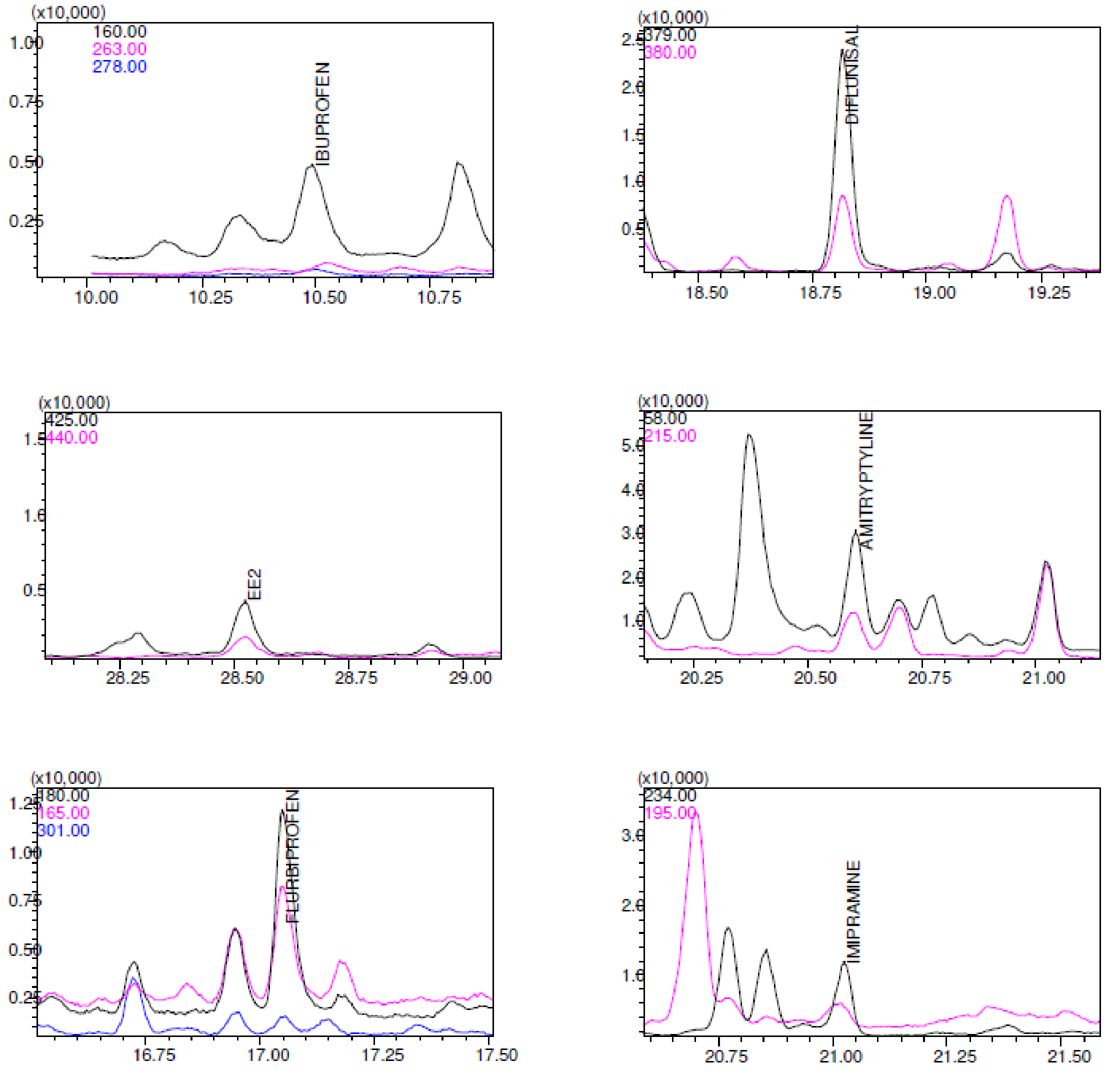

Figure S3 Example chromatogram with marked SIM ions for determined target compounds in real Papyrus (*Cyperus papyru*) samples

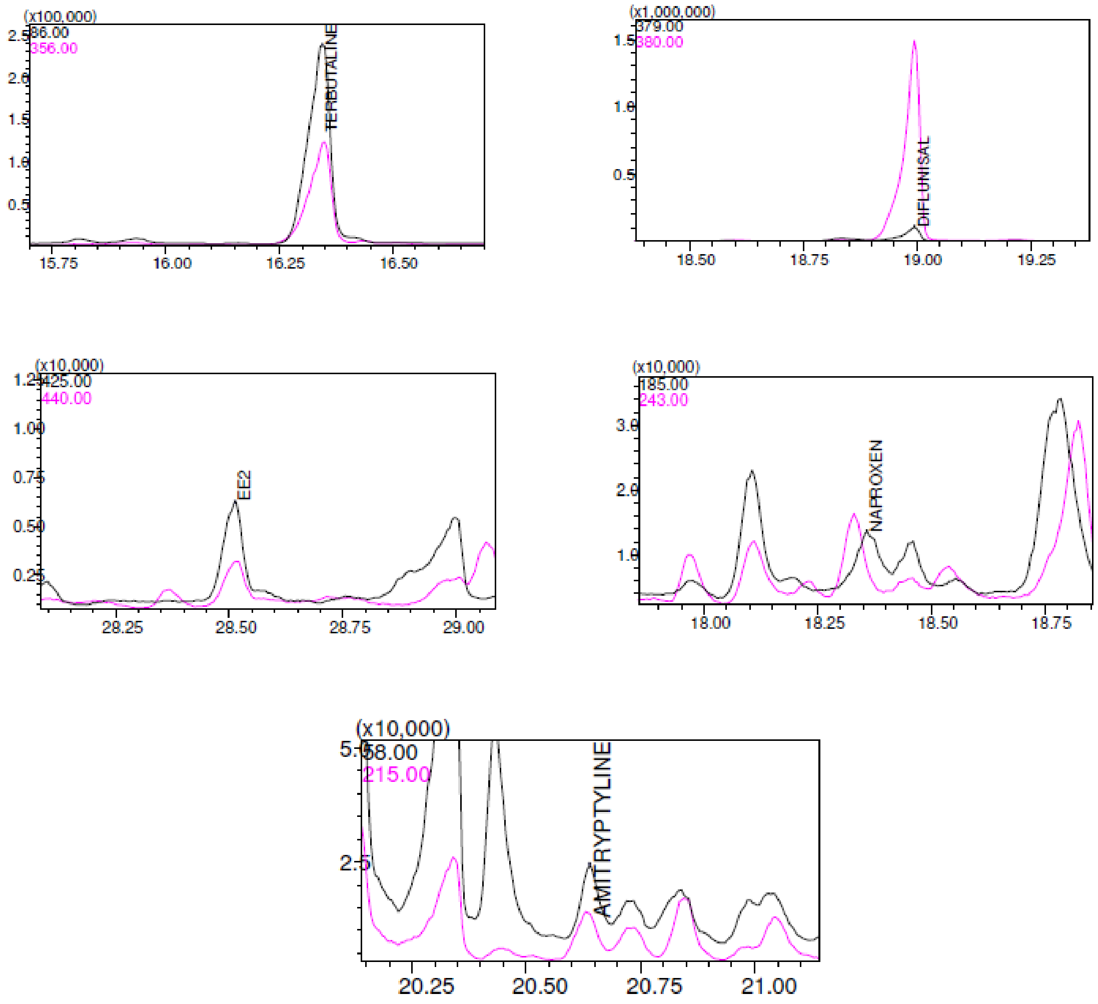

Figure S4 Example chromatogram with marked SIM ions for determined target compounds in real Yellow pimpernel (*Lysimachia nemorum*) samples

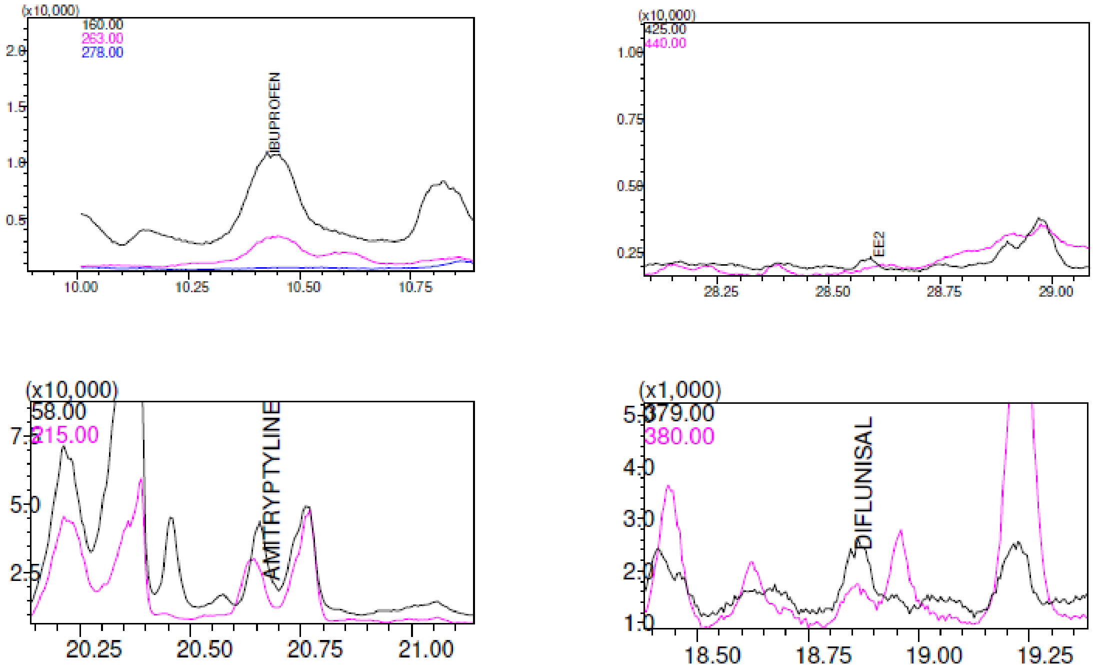

Figure S5 Example chromatogram with marked SIM ions for determined target compounds in real European spindle (*Euonymus europaeus*) samples

© 2019 by the authors. Submitted for possible open access publication under the terms and conditions of the Creative Commons Attribution (CC BY) license (http://creativecommons.org/licenses/by/4.0/).
